# Cytomegalovirus infections in thoracic organ transplant recipients: Updates on prevention, treatment, and immune monitoring

**DOI:** 10.1016/j.jhlto.2025.100449

**Published:** 2026-01-16

**Authors:** Brennan J. Collis, Madeleine R. Heldman, Cameron R. Wolfe

**Affiliations:** Division of Infectious Diseases, Department of Medicine, Duke University School of Medicine, Durham, NC

**Keywords:** Cytomegalovirus, Heart transplant, Lung transplant, Prevention, Treatment, Cell-mediated immunity

## Abstract

**Background:**

Cytomegalovirus (CMV) is a major cause of morbidity following solid organ transplantation, with thoracic organ transplant recipients (TOTRs) representing one of the highest risk groups. Despite this elevated risk, TOTRs are under-represented in clinical research, and current management strategies are largely extrapolated from other transplant populations.

**Methods:**

This review synthesizes evidence on CMV epidemiology, clinical outcomes, prevention, and treatment in TOTRs.

**Key content:**

Despite the success of antiviral prophylaxis and pre-emptive monitoring strategies in reducing CMV-related complications in this population, late-onset infection and antiviral resistance remain major clinical challenges. We explore the potential role of CMV in chronic rejection, evaluate the utility of CMV cell-mediated immune monitoring, and review the clinical experience with novel antivirals in TOTRs. By identifying key evidence gaps and outlining priorities for future research, this review aims to support the development of targeted and more effective CMV management strategies in this high-risk population.

## Introduction

Despite recent advances, cytomegalovirus (CMV) remains a major cause of morbidity in thoracic organ transplant recipients (TOTRs).^1^ Lung transplant recipients (LTRs) are at particularly high risk for CMV infections. CMV risk in heart transplant recipients (HTRs) is generally higher than in liver or kidney recipients but lower than in LTRs.^2^

Managing CMV in TOTRs presents unique challenges not fully addressed by general transplant guidelines which are largely informed by kidney, liver and allogeneic hematopoietic stem cell transplant (HSCT) recipient data.^3^, ^4^ Given organ-specific aspects of CMV biology and heightened immunosuppression in TOTRs, a nuanced approach to prevention, diagnosis, and treatment is essential. This review outlines the current understanding of CMV in adult TOTRs, highlighting population-specific evidence, clinical challenges, and priorities for future research to improve outcomes in this high-risk group.

### Pathophysiology

CMV is a β-herpesvirus, with adult seroprevalence ranging from 60%−90% worldwide.^5^

Primary infection typically occurs in childhood. In immunocompetent hosts, CMV triggers robust innate and adaptive immune responses, particularly CMV-specific CD8+ T-cells, leading to rapid control of viral replication and an asymptomatic/mild illness.^6^ CMV then establishes lifelong latency in CD34+ hematopoietic progenitor and endothelial cells, with potential for reactivation.^7^, ^8^ In immunocompromised individuals, impaired immunity permits viral replication, resulting in DNAemia and/or tissue-invasive disease. Infection in solid organ transplant recipients (SOTRs) may arise from primary infection in CMV-naïve individuals, reactivation of latent virus acquired before transplant, or superinfection with a novel strain. Primary infection or superinfection in SOTRs are frequently transmitted through allografts from CMV seropositive donors.

### Risk factors

Risk factors for post-transplant CMV infection in TOTRs mirror those observed in other SOTRs.^4^ The most predictable risk is donor-recipient serostatus. Seronegative recipients of seropositive donor organs (D+R-) are at highest risk due to lack of pre-existing immunity. Seropositive recipients (R+) are at intermediate risk, while seronegative recipients of seronegative donor organs (D-R-) face the lowest risk. Most other established risk factors are shared between HTRs and LTRs, though several are unique to each group (Table 1).

**Table 1:** Reported Risk Factors Associated with Cytomegalovirus Infection in Thoracic Organ Transplant Recipients

Organ Type	Risk Factor	Reference
Both lung and heart transplant recipients	CMV serostatus	23, 118, 119, 120, 121
	Graft rejection	^122^
	Use of lymphodepleting immunosuppressive agents	121, 123
	Hypogammaglobulinemia within the first year post-transplant	^124^
	Truncated antiviral prophylaxis duration	121, 125
	Lymphopenia	23, 48, 126, 127
	Maintenance immunosuppression regimens that do not contain a mTOR inhibitor	128, 129
Heart transplant recipients	Ciclosporin versus tacrolimus containing maintenance immunosuppression regimens	^130^
	Mycophenolate versus azathioprine containing maintenance immunosuppression regimens	^131^
Lung transplant recipients	Advanced donor age	125, 132
	Pre-operative hypoalbuminemia	^132^

Abbreviations: mTOR, mammalian target of rapamycin.

LTRs have a particularly high risk of CMV infection due to the lung allograft’s extensive surface area and high concentration of lymphoid tissue and endothelial cells, which require intensive immunosuppression protocols to prevent rejection. The high concentration of lymphoid cells also carries a large reservoir of CMV in lung allografts from CMV seropositive donors.^9^ HTRs have a lower risk of CMV infection than LTRs, but may be at higher risk than abdominal organ recipients due to relatively high immunosuppression requirements.^10^ The widespread use of antiviral prophylaxis (AP) has significantly reduced the incidence of early post-transplant CMV infection in both HTRs and LTRs, but CMV infection is common after stopping AP, especially in CMV D+R- TOTRs.

### Clinical manifestations

CMV infection in SOTRs ranges from asymptomatic DNAemia to life-threatening end-organ disease. CMV DNAemia is defined as detectable viral DNA in blood/serum.^11^ DNAemia may occur without CMV disease (“asymptomatic DNAemia”) or with CMV disease. CMV disease includes end-organ disease or CMV syndrome, and standardized definitions are recommended for characterizing CMV disease outcomes in research settings.^11^CMV disease is the preferred clinical endpoint for prevention trials by the U.S Food and Drug Administration (FDA).^12^ CMV disease is more common in D+R- TOTRs than R+ TOTRs and most frequently occurs within six months after cessation of AP.

### Diagnosis

#### CMV DNAemia

Quantitative polymerase chain reaction (qPCR) testing of blood/plasma for CMV DNA is a standard method for diagnosis of CMV infection.^11^ However, DNAemia does not always correlate with disease,^13^, ^14^ and inter-laboratory variability limits assay comparability across sites.^15^

#### CMV syndrome

CMV syndrome is a clinical diagnosis characterized by DNAemia, systemic symptoms, and laboratory abnormalities.^11^ It typically occurs during primary infection in CMV seronegative organ recipients, usually after antiviral prophylaxis cessation in D+R- individuals, but may also occur in community-acquired infection in D-R- patients.

#### CMV end-organ disease

Diagnosis of end-organ CMV disease requires clinical symptoms with histopathological confirmation, including viral inclusions or positive immunohistochemical staining.^11^ While research definitions require tissue to document probable or proven end-organ disease (retinitis excepted), clinical management may not hinge on establishing a diagnosis of end-organ tissue. For example, in an individual with CMV syndrome, high level CMV DNAemia, and diarrhea are suggestive of CMV colitis, but establishing a diagnosis of proven/probable CMV gastrointestinal disease does not necessarily impact the approach to antiviral therapy. While CMV DNAemia correlates with end-organ disease risk, certain manifestations like enteritis,^16^ retinitis,^16^ and pneumonitis^17^ may occur despite low/undetectable DNAemia, especially in seropositive SOTRs.

CMV pneumonitis is uncommon among SOTRs and occurs predominately in D+/R- LTRs.^17^ Lung biopsy remains the diagnostic gold standard and is frequently performed in LTRs. Quantification of CMV DNA in bronchoalveolar lavage (BAL) fluid has limited diagnostic utility in this population as the lung serves as a major reservoir of latent CMV and viral shedding may occur secondary to tissue injury of inflammation from other causes.^18^ Furthermore, no validated threshold has been developed to reliably differentiate CMV pneumonitis from asymptomatic shedding in SOTRs, including in LTRs.^11^ Variability in sampling techniques, assay methods, and the focal nature of CMV lesions further complicate interpretation. Consequently, many transplant centers do not routinely perform or apply defined diagnostic cutoffs for BAL CMV DNA quantification.

### Outcomes

#### Mortality

CMV disease has a well-established association with mortality in TOTRs.^19^, ^20^ However, direct CMV-attributable deaths are uncommon and the mechanism underlying this association is incompletely understood. The advent of highly sensitive assays has enabled the detection of low-level DNAemia, which may reflect latent CMV virions rather than active viral replication. Studies have yielded mixed findings regarding a correlation between asymptomatic DNAemia and mortality.^21^, ^22^, ^23^, ^24^ Controlling for potential confounders between CMV DNAemia and mortality is often challenging and it is not clear whether CMV DNAemia lies on this causal pathway. CMV DNAemia also occurs in critically ill immunocompetent individuals and may reflect an “innocent bystander” rather than a pathogen necessitating treatment in certain settings.^25^

Notably, D+R- TOTRs, and to a lesser extent R+ TOTRs, have worse survival compared to D-R- patients.^23^, ^26^, ^27^ These disparities become more evident after the first transplant year, when most CMV episodes have occurred, suggesting that persistent CMV replication within the allograft may contribute to chronic injury and impaired long-term survival, even in the absence of overt DNAemia or disease.

#### Chronic allograft vasculopathy (CAV)

The role of CMV in the pathogenesis of CAV in HTRs is debated. Early randomized clinical trials (RCTs) demonstrated reduced CMV-related graft failure with AP,^28^, ^29^ and early observational data linked CMV infection to coronary artery disease or intimal thickening (Table 2).^30^, ^31^, ^32^, ^33^ However, later observational studies yielded inconsistent results,^34^, ^35^, ^36^ likely reflecting heterogeneity in CMV/CAV definitions and follow-up durations.

**Table 2:** Observational Studies Examining the Association Between Post-transplant Cytomegalovirus Infection and Chronic Rejection Phenotypes in Thoracic Organ Transplant Recipients

Study	Country	Study Type	Sample Size	Study Period	Validated Definition for CMV Infection	Validated Definition for Chronic Rejection	Follow-up (Median/Mean)	Major Finding
Association between post-transplant CMV infection and CAV in heart transplant recipients								
Grattan et al., JAMA 1989^30^	USA	Retrospective single center	301	1980-1988	No	No	Not reported	CMV infection (defined by seroconversion, positive viral culture, or inclusion body on histopathological assessment) was significantly associated with atherosclerosis up to 3 months post-transplant.
McDonald et al., Am J Cardiol 1989^31^	USA	Retrospective single center	102	1983-1987	No	No	1.0 years	CMV infection (defined by seroconversion combined with a positive viral culture) was significantly associated with coronary artery disease.
Sharples et al., Transplantation 1991^133^	UK	Retrospective single center	323	1979-1989	No	No	Not reported	CMV infection (defined by seroconversion, positive viral culture or histology suggestive of end organ disease) was not associated with the development of coronary occlusive disease.
Koskinen et al., Transpl Int 1993^32^	Finland	Retrospective single center	53	Not reported	No	No	Not reported	CMV infection (defined as positive IgM serology, positive viral culture combined with a four-fold rise in CMV IgG titer, or antigenemia) was associated with the development of discrete stenoses in major branch vessels, higher levels of arteriolar endothelial cell proliferation, and higher intimal thickness.
Potena et al., Transplantation 2003^33^	Italy	Prospective observational study	29	1998-2000	No	No	Not reported	Patients with CMV antigenemia that required treatment was associated with impaired ability of the coronary vessel wall to enlarge in response to intimal volume increase (i.e. lumen loss).
Sharples et al., Transplantation 2003^134^	UK	Retrospective single center	566	1979-2002	No	No	Not reported	CMV infection (not otherwise defined) was significantly associated with the development of CAV.
Petrakopoulou et al., Circulation 2004^135^	Germany	Retrospective analysis of a prospective study	183	Not reported	No	No	5.5 years	CMV infection (defined by pp65 antigenemia) was significantly associated with microvascular endothelial dysfunction.
Johansson et al., BMC Infect Dis 2015^20^	Sweden	Retrospective single center	278	1988-2000	Yes^136^	Yes^137^	8.9 years	CMV infection (defined by CMV detected by viral culture or PCR in any body fluid or tissue specimen) and CMV disease were associated with reduced CAV-free survival.
Delgado et al., J Heart Lung Transplant 2015^138^	Spain	Retrospective analysis of a prospective study	166	1995-2002	Yes^139^, ^140^	Yes^141^	11.0 years	CMV infection (defined by positive antigenemia) and CMV disease were associated with the development of CAV.
Galli et al., Neth Heart J 2016^142^	Netherlands	Retrospective single center	295	1984-2012	Not reported	Yes^141^	10.6 years	CMV disease (not otherwise defined) was not associated with a significant increase in CAV.
Goekler et al., Transpl Int 2018^34^	Austria	Retrospective single center	297	2002-2012	No	Yes^143^	7.5 years	CMV infection (defined as CMV DNAemia >1000 copies/mL) was not significantly associated with an increased risk of CAV.
Klimczak-Tomaniak et al., Transplantation 2020^35^	Netherlands	Retrospective single center	260	2000-2018	Yes^144^	Yes^141^	7.9 years	No significant association was observed between CMV infection (defined as CMV DNAemia >1000 IU/mL with/without symptoms or <1000 IU/mL with symptoms) and CAV, except for patients with breakthrough CMV infection.
Das et al., Pediatr Transplant 2020^36^	USA	Retrospective single center	97 (Pediatric)	2010-2016	Yes^144^	Yes^145^	3.0 years	The detection of CMV by indirect immunofluorescent assay on endomyocardial biopsy was not associated with an increased risk of CAV compared to patients who had CMV DNAemia alone.
Association with CLAD (BOS, OB) in lung transplant recipients								
Keenan et al., Transplantation 1991^37^	USA	Retrospective single center	27	1986-1989	No	No	Not reported	Post-transplant CMV disease (defined by inclusion bodies on biopsy or culture of CMV from BAL fluid) was not associated with OB.
Cerrina et al., Transpl Int 1992^38^	France	Retrospective single center	36	1986-1990	No	No	2.0 years	OB occurred more frequently in patients with CMV pneumonitis (defined by the presence of CMV on BAL/TBB, inclusion bodies on TBB, and lung infiltrates on chest radiograph) or post-transplant CMV infection (defined by the presence of CMV in lung, blood, and/or seroconversion).
Ettinger et al., Am Rev Respir Dis 1993^39^	USA	Retrospective single center	50	1988-2000	No	Not reported	1.2 years	Post-transplant CMV infection (defined as CMV in blood or BAL fluid as detected by viral culture) was not associated with the development of BOS.
Bando et al., J Thorac Cardiovasc Surg 1995^40^	USA	Retrospective single center	162	1982-1992	No	Yes^146^, ^147^	5.0 years	CMV pneumonitis (defined as positive CMV culture and the presence of intracellular inclusions typical of CMV in cells or tissues obtained from lung tissue) was associated with the development of OB on univariate but not multivariate analysis.
Kroshus et al., J Thorac Cardiovasc Surg 1997^41^	USA	Retrospective single center	132	1986-1995	No	Yes^146^, ^147^	Not reported	Symptomatic CMV pneumonitis (defined as a new pulmonary infiltrate, compatible symptoms, and new CMV culture positivity or cytological evidence of CMV inclusions on BAL or histopathology) was a significant predictor of BOS and OB.
Gutierrez et al., Chest 1998^42^	Canada	Retrospective single center	56	1993-1995	No	Yes^147^	1.8 years	CMV infection (defined as pp65 antigenemia, blood CMV viral-shell culture, or BAL viral-shell culture or antigen detection by immunofluorescence), recipient serology at transplant, and CMV disease were not associated with the development of BOS.
Husain et al., Am J Respir Crit Care Med 1999^43^	USA	Retrospective single center	134	1990-1995	No	Yes^147^	2.3 years	CMV pneumonitis (not otherwise defined) was not associated with the development of BOS.
Westall et al., Transplantation 2003^148^	Australia	Retrospective single center	23	1997-1998	Yes^136^	Yes^149^	3.1 years	CMV DNAemia >10 copies/µg total DNA was associated with the development of BOS, but CMV pneumonitis was not associated with development of BOS.
Tamm et al., Am J Respir Crit Care Med 2004^150^	Australia	Retrospective single center	341	1987-2001	No	Yes^149^	Not reported	CMV pneumonitis (defined by the presence on TBB of typical inclusion bodies with cytopathic effect) was not associated with the development of BOS at 1, 3 and 5 years post-transplant.
Valentine et al., J Heart Lung Transplant 2009^151^	USA	Retrospective single center	161	1990-2005	No	Yes^147^	4.3 years	CMV pneumonitis within the first 100 days post-transplant was associated with the development of BOS.
Thomas et al., Clin Transplant 2009^152^	USA	Retrospective single center	78	1994-2000	No	No	4.3 years	Post-transplant CMV infection (defined as positive culture from any tissue or body fluid or a positive blood CMV antigenemia) was associated with BO and BOS.
Manuel et al., Transplantation 2009^153^	USA	Retrospective single center	93	2003-2005	CMV disease only^154^	Yes^149^	2.1 years	Detection of BAL CMV PCR>100 copies/mL was not associated with the development of OB/BOS.
Snyder et al., Am J Respir Crit Care Med 2010^155^	USA	Retrospective single center	231	2000-2004	No	Yes^147^	6.7 years	Treated CMV pneumonitis (defined as positive CMV immunohistological staining on TBB) within 6 months post-transplant significantly increased the risk for the development of BOS.
Mitsani et al., J Heart Lung Transplant 2010^156^	USA	Retrospective single center	109	2003-2008	No	Not reported	2.3 years	Post-transplant CMV infection (not otherwise defined) and CMV disease were not associated with the development of BOS.
Johansson et al., Scand J Infect Dis 2010^157^	Sweden	Retrospective single center	187	1990-2002	Yes^136^	No	Not reported	CMV pneumonitis was associated with a significant increased risk of developing BOS at 1 and 2 years post-transplant.
Paraskeva et al., Am J Transplant 2011^158^	Australia	Retrospective single center	192	2001-2009	No	Yes^147^, ^149^	2.8 years	Post-transplant detection of CMV DNA in BAL supernatant was significantly associated with an increased risk of BOS.
Verleden et al., Transplantation 2013^159^	Belgium	Retrospective single center	380	2001-2011	No	No	4.5 years	CMV pneumonitis (not otherwise defined) was not associated with the development of BOS or RAS.
Johansson et al., BMC Infect Dis 2013^160^	Sweden	Retrospective single center	114	2001-2006	Yes^136^	No	Not reported	CMV pneumonitis, but not asymptomatic CMV infection (either CMV DNA detected in serum or positive immunohistochemistry on TBB or BAL), was associated with reduced BOS 4 year survival.
Fisher et al., Clin Infect Dis 2016^161^	USA	Retrospective single center	250	2007-2012	No	Yes^149^, ^162^	2.7 years	CMV pneumonitis (defined as positive shell vial and/or positive viral culture from a BAL with compatible symptoms and radiographic findings) was not associated with the development of CLAD.
Monforte et al., Transpl Infect Dis 2017^125^	Spain	Prospective multicentre observational study	92	2007-2009	Yes^136^	No	1.5 years	CMV pneumonitis was not associated with the development of CLAD.
Jaamei et al., Transpl Infect Dis 2018^44^	Switzerland	Retrospective single center	69	2004-2014	Yes^163^	Yes^162^, ^164^, ^165^	3.7 years	Post-transplant significant CMV infection (defined as 2x consecutive blood CMV DNA >100 copies/mL or 1x blood CMV DNA >1000 copies/mL), duration of CMV replication, and CMV pneumonitis were not associated with CLAD.
Chang et al., Transpl Infect Dis 2019^21^	Australia	Retrospective single center	137	2004-2017	Yes^144^	Yes^149^	4.1 years	CMV viremia (not otherwise defined) was not associated with the development of BOS.
Bennett et al., Lung 2022^166^	Italy	Retrospective single center	87	2012-2021	NA	Not reported	Not reported	Post-transplant CMV DNAemia >10000 copies/mL was associated with a significantly shorter CLAD-free survival.
Kawashima et al., Am J Transplant 2024^22^	Canada	Retrospective single center	647	2010-2016	NA	Yes^162^	Not reported	CMV DNAemia >10000 IU/mL was not associated with the development of CLAD.

Orange box denotes studies that identified a significant association between post-transplant CMV infection and chronic rejection. Green box denotes studies that did not identify a significant association.

Abbreviations: BAL, bronchoalveolar lavage; BO, bronchiolitis obliterans; BOS, bronchiolitis obliterans syndrome; CAV, chronic allograft vasculopathy; CLAD, chronic lung allograft dysfunction; CMV, cytomegalovirus; DNA, deoxyribonucleic acid; IgG, immunoglobulin G; NA, not applicable; OB, obliterative bronchiolitis; PCR, polymerase chain reaction; TBB, trans-bronchial biopsy.

#### Chronic lung allograft dysfunction (CLAD)

Similarly, evidence linking CMV and CLAD in LTRs is inconclusive. Early studies showed mixed results and often did not adjust for serostatus.^37^, ^38^, ^39^, ^40^, ^41^, ^42^, ^43^ Contemporary studies using standardized prophylaxis and immunosuppression regimens have not identified CMV DNAemia or disease to be consistent predictors of CLAD (Table 2).^21^, ^22^, ^44^ Notably, a recent large retrospective cohort study of 647 LTRs receiving 6–9 months of AP found D+R- serostatus, but not DNAemia, to be associated with increased CLAD risk.^22^ This suggests DNAemia may not fully capture localized CMV replication in the lung allograft or that the mechanisms behind poor outcomes in CMV D+R- LTRs are not directly related to CMV replication.

### Prevention

#### Antiviral prophylaxis versus pre-emptive therapy

CMV disease in SOTRs can be effectively prevented in the early post-transplant period with either AP or pre-emptive therapy (PET). AP involves administering antivirals to at-risk patients for a predetermined duration, whereas PET entails routine CMV DNAemia monitoring, with treatment initiated upon exceeding a predefined threshold. Universal AP is easier to implement but puts all patients at risk of adverse effects (e.g. myelotoxicity) and may impair development of CMV-specific immunity. PET allows immune priming through controlled subclinical replication, which is particularly important for long-term protection in D+R- patients. PET also limits antiviral exposure to only those with DNAemia, often allowing lower risk patients (e.g. R+ individuals) to avoid antiviral exposure altogether. However, PET is logistically complex and prone to delayed detection if monitoring fails, increasing the risk of CMV disease, particularly in D+R- patients who exhibit rapid CMV viral kinetics.

In kidney and liver transplant recipients, both AP and PET are accepted standards of care for both D+R- and R+ patients.^3^ However, in TOTRs, no RCTs have directly compared these strategies for preventing CMV disease or evaluating long-term outcomes. Current guidelines recommend AP for all D+R- TOTRs due to their elevated CMV risk, rapid replication kinetics, and the absence of data supporting the efficacy or graft-protective benefits of PET).^3^ In R+ LTRs, AP is preferred due to limited data supporting PET and ongoing concerns regarding the indirect effects of permissive DNAemia on graft outcomes. In R+ HTRs, observational data supports the safety of PET,^45^, ^46^, ^47^, ^48^ though AP has been associated with lower CMV infection and improved graft outcomes in some observational studies.^49^, ^50^

#### Antiviral prophylaxis

Ganciclovir and valganciclovir are first-line agents for CMV prophylaxis in TOTRs (Table 3), though their use is limited by myelosuppression and need for renal dose adjustment.^3^

**Table 3:** Recommended Post-transplantation Cytomegalovirus Prophylaxis Strategies for Thoracic Organ Transplant Recipients, Stratified by Risk Level

CTR	CMV Serostatus	Risk Level	Prophylaxis		
			Preferred Regimen	Alternate Regimen	Notes
Lung	D+R-	High	At least 12 months of IV ganciclovir (5mg/kg q24h) or PO valganciclovir (900 mg q24h)^a^.	At least 12 months of PO letermovir (480 mg q24h)^b^. If unable to tolerate or access (val)ganciclovir or letermovir, then consider PET with q1 weekly CMV qPCR testing and initiation of antiviral therapy (ganciclovir or valganciclovir) if CMV DNA >2-3.2 log_10_ IU/mL i.e.100-1500 IU/mL. Consider adjunctive CMVIG.	RCT data: 12 months AP was superior to 3 months for prevention of CMV disease.^63^ Observational data: 9 months AP was associated with a lower incidence of CMV disease than 6 months AP.^64^
	R+	Intermediate	6-12 months of IV ganciclovir (5mg/kg q24h) or PO valganciclovir (900 mg q24h)^a^.	6-12 months of PO letermovir (480 mg q24h)^b^. If unable to tolerate or access (val)ganciclovir or letermovir, then consider PET with initiation of antiviral therapy (ganciclovir or valganciclovir) if CMV DNA >2.7-3.6 log_10_ IU/mL i.e. 500-4000 IU/mL).	RCT data: 12 months AP is superior to 3 months for prevention of CMV disease.^63^ Observational data: 6 months of AP in D-R+ was associated with a lower incidence of CMV DNAemia or disease than 6 months AP in D+R- or D+R+.^65^
	D-R-	Low	CMV prophylaxis not recommended	NA	NA
Heart	D+R-	High	3-6 months of IV ganciclovir (5mg/kg q24h) or PO valganciclovir (900 mg q24h)^a^.	3-6 months of PO letermovir (480 mg q24h)^b^. If unable to tolerate or access (val)ganciclovir or letermovir, then consider PET with q1 weekly CMV qPCR testing and initiation of antiviral therapy (ganciclovir or valganciclovir) if CMV DNA >2-3.2 log_10_ IU/mL i.e.100-1500 IU/mL). Consider adjunctive CMVIG.	Observational data: no difference in CMV disease incidence with ≥12 months and ≤6 months of AP.^67^ Observational data: 3 months AP was associated with a similar incidence of CMV disease as 6 months AP.^68^
	R+	Intermediate	3 months of IV ganciclovir (5mg/kg q24h daily) or PO valganciclovir (900 mg q24h)^a^, or, PET with initiation of antiviral therapy (ganciclovir or valganciclovir) if CMV DNA >2.7-3.6 log_10_ IU/mL i.e. 500-4000 IU/mL.	3 months of PO letermovir (480 mg daily)^b^.	Observational data: no difference in CMV disease incidence with ≥12 months and ≤6 months of AP.^67^
	D-R-	Low	CMV prophylaxis not recommended	NA	NA

Abbreviations: AP, antiviral prophylaxis; CMV, cytomegalovirus; CTR, cardiothoracic recipient; D, donor; DNA, deoxyribonucleaic acid;IU, international units; IV, intravenous; mL, milliliters; NA, not applicable; PET, pre-emptive therapy; PO, per os; qPCR, quantitative polymerase chain reaction; R, recipient; RCT, randomized clinical trial.

a assumes normal renal function for ganciclovir and valganciclovir.

b assumes lack of co-administered medications necessitating a lower prescribed dose of letermovir (e.g. cyclosporine).

Letermovir, a viral terminase complex inhibitor, is approved by the US FDA for CMV prophylaxis in R+ allogeneic HSCT and D+R- kidney recipients.^51^ While RCTs in TOTRs are lacking, there is emerging observational data supporting its safety and efficacy in both D+R- and R+ TOTRs with (val)ganciclovir intolerance/resistance.^52^, ^53^, ^54^, ^55^, ^56^, ^57^, ^58^, ^59^, ^60^ A recent prospective study of 32 D+R- and R+ HTRs also demonstrated comparable clinical effectiveness and fewer cases of neutropenia with letermovir primary prophylaxis when compared to a predominately historical valganciclovir prophylaxis cohort.^61^ A similar study is underway comparing a mixed LTR and HTR cohort to a historical valganciclovir population(NCT06066957). In response to this emerging evidence, some experts consider letermovir first-line in select LTRs at high risk of valganciclovir toxicity, particularly those with renal insufficiency, advanced age, high risk of rejection, or receiving cytolytic immunosuppression, though cost and access remain barriers.^53^, ^62^

Optimal AP duration in TOTRs remains undefined. In LTRs, a RCT demonstrated 12 months of valganciclovir was superior to 3 months in preventing CMV disease through day 300 in both D+R- and R+ patients.^63^ A subsequent retrospective study demonstrated 9 months AP was associated with less CMV disease than a 6-month duration in D+R- LTRs.^64^ In D-R+ LTRs, 6 months of AP was associated with a significantly lower incidence of CMV DNAemia/disease than in D+R- or D+R+ individuals who received the same duration.^65^ Guidelines therefore recommend 6 months AP in R+ and 6–12 months in D+R- LTRs.^3^, ^4^

In D+R- LTRs, CMV disease frequently occurs following AP cessation and therefore some centers have adopted lifelong prophylaxis to reduce CMV disease incidence, though cumulative toxicity, cost, and resistance remain concerns.^19^, ^66^

Evidence guiding AP duration in HTRs is limited. A small retrospective study found no difference in CMV disease risk when comparing ≤6 to ≥12 month durations in both D+R- and R+ HTRs.^67^ Other data suggest no difference in CMV disease incidence after 3-months vs. 6-months of AP in D+R- HTRs.^68^ Current guidelines recommend 3-months in R+ HTRs based on expert opinion and extrapolation from other organ transplant cohorts.^3^, ^4^

Post-prophylaxis strategies vary by center, organ, and serostatus. They include discontinuation with symptom-based testing only, or CMV DNAemia surveillance. CMV-specific cell-mediated immune (CMV-CMI) monitoring is a novel strategy to identify individuals expected to benefit from additional prophylaxis or closer monitoring (see Section 2.8).

#### CMV immune globulin (CMVIG)

CMVIG is a purified antibody product derived from pooled donor plasma. It contains significantly higher titers of CMV-specific and neutralizing antibodies than standard intravenous immunoglobulin (IVIG) and has been evaluated in the prevention and management of CMV infection in transplant recipients.^69^, ^70^

The evidence supporting CMVIG for CMV prophylaxis in TOTRs is limited to observational data. Early studies, conducted before routine AP implementation, reported a reduced incidence of CMV infection with CMVIG compared to placebo.^71^, ^72^, ^73^, ^74^ Later studies, evaluating CMVIG plus AP versus AP alone, yielded similar findings,^75^, ^76^, ^77^, ^78^ although the duration of AP used was often shorter than current guideline recommendations. To date, no prospective interventional trials have assessed CMVIG plus AP against contemporary AP regimens. In the absence of high-quality evidence, CMVIG prophylaxis is not routinely recommended by clinical guidelines.^3^, ^4^ Nonetheless, surveys conducted in 2009 and 2011 indicated that 32–38% of transplant centers use adjunctive CMVIG in addition to AP in D+R- TOTRs.^79^, ^80^

#### Mammalian target of rapamycin (mTOR) inhibitors

In SOTRs, mTOR inhibitors have been associated with a reduced incidence of CMV disease, likely through their dual effects on enhancing T-cell function and inhibiting CMV replication.^81^ In D+R- and R+ HTRs, evidence suggests that the combination of mTOR inhibitors with 100 days of AP is associated with lower rates of CMV infection compared to AP or PET alone,^82^ supporting its consideration in patients with recurrent CMV infection or at high risk of CMV disease. However, this must be considered against the potential toxicities of mTOR inhibitors in the early postoperative period, including effusions and delayed wound healing.

### Management

**Figure 1:**
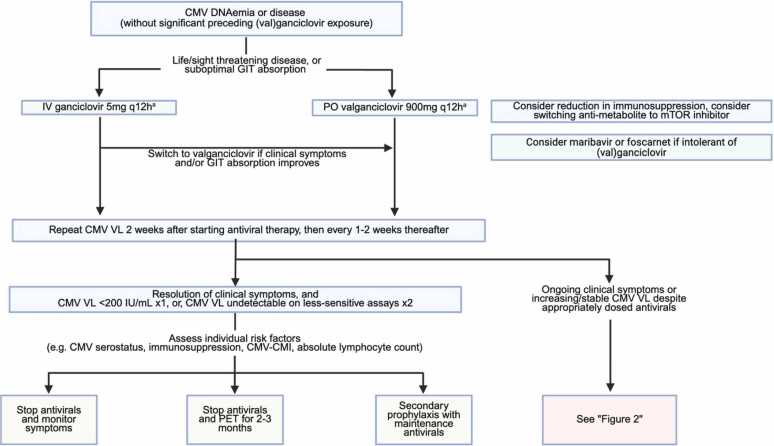
Management algorithm for post-transplant cytomegalovirus infection in thoracic organ transplant recipients. ^*a*^*assumes normal renal function. Abbreviations: CMV, cytomegalovirus; CMV-CMI, cytomegalovirus cell-mediated immunity; GCV, ganciclovir; GIT, gastrointestinal; IV, intravenous; mTOR, mammalian target of rapamycin; PET, pre-emptive therapy; PO, per os; (v)GCV, valganciclovir or ganciclovir; VL, viral load.*

The management of CMV infection in TOTRs follows the same principles established in other SOTRs (Fig. 1). Core strategies include immunosuppression reduction when feasible and initiation of antiviral therapy. For non-life-threatening disease, oral valganciclovir is non-inferior to intravenous ganciclovir as shown in a RCT of 321 SOTRs, including 37 TOTRs.^83^ Intravenous ganciclovir is preferred in life-threatening disease or gastrointestinal involvement limiting oral absorption.^3^, ^4^ Antiviral therapy should continue until clinical resolution and eradication of DNAemia. Post-treatment management, however, remains poorly defined. Common practices include secondary prophylaxis or antiviral discontinuation with clinical monitoring and/or DNAemia surveillance. No interventional trials have compared these strategies in TOTRs or SOTRs. Although secondary prophylaxis is widely used, retrospective data from mixed SOTR cohorts do not demonstrate reduced rates of recurrent CMV disease.^84^, ^85^, ^86^, ^87^, ^88^ Post-treatment management should therefore be individualized. Clinicians should consider the risk of recurrence (determined by CMV serostatus, organ transplant type, and net state of immunosuppression), as well as the patient’s overall health, capacity to tolerate recurrent infection, and practical factors such as access to laboratory monitoring and antiviral cost. Given the higher relapse risk among TOTRs, we consider secondary antiviral prophylaxis for one to three months in high-risk patients (e.g., D+R-) in an attempt to facilitate greater immunologic recovery before antiviral discontinuation, while acknowledging the limited evidence supporting this approach.

#### CMV immune globulin

Beyond its role in prophylaxis, CMVIG is sometimes used as adjunctive therapy for the treatment of CMV DNAemia or disease. The strongest evidence supporting this practice originates from studies in allogeneic HSCT recipients with CMV pneumonitis in the 1980s.^89^ In TOTRs, evidence is limited to several case series and a small observational study,^90^ with substantial heterogeneity in indications, dosing, and concurrent antiviral regimens.^91^ CMVIG may be considered in select TOTRs with severe disease, refractory/resistant infections, antiviral intolerance, or concurrent hypogammaglobulinemia, although these recommendations are based on expert opinion.^3^, ^4^

#### Refractory/resistant (R/R) CMV infection

Refractory CMV infection is defined by increasing or persisting DNAemia after ≥2 weeks of appropriately dosed antiviral therapy.^11^ Resistance is identified by genotypic mutations that confer reduced antiviral susceptibility (Table 4). Risk factors for R/R CMV infection include LTRs, D+R- serostatus, prolonged antiviral exposure, and subtherapeutic antiviral dosing.^92^, ^93^, ^94^ As such, D+R- LTRs receiving extended/lifelong AP represent the highest risk group. Incidence of R/R CMV is approximately 5% in D+R- TOTRs and less common in R+ recipients.^95^, ^96^ R/R infection is associated with increased mortality in TOTRs.^92^, ^95^

**Table 4:** Common Mutations Associated with Resistance to Cytomegalovirus Antivirals

Drug	Gene	Mutation	Fold-increase in EC_50_	Cross-resistance
Ganciclovir	UL97	M460V	8.3	-
		M460I	5	-
		H520Q	10	-
		C592G	2.9	-
		L595S	9.2	-
		L595F	15.7	-
		C603W	8	-
Maribavir	UL97	F342Y	4,7	Ganciclovir reduced susceptibility
		H411Y	17	-
		C480F	>200	Ganciclovir reduced susceptibility
		T490M	80	-
Letermovir	UL56	C325F	>3000	-
		C325Y	>3000	-
		C325R	>3000	-
		C325W	>3000	-
Foscarnet	UL54	D588E	2.3	-
		D588N	3.2-9	Ganciclovir and cidofovir reduced susceptibility
		T700A	4.7	-
		V715M	5.5	-
		E756D	3.4	-
		E756K	>8	Ganciclovir and cidofovir reduced susceptibility
		E756Q	4.3	-
		L776M	3.5	Ganciclovir reduced susceptibility
		V781L	4-5.2	Ganciclovir reduced susceptibility
		V787L	4.1	Ganciclovir reduced susceptibility
		L802M	3.2-10.8	Ganciclovir reduced susceptibility
		A809V	6.3	Ganciclovir reduced susceptibility
		V812L	2.9	Ganciclovir and cidofovir reduced susceptibility
		T813S	4.9	Ganciclovir reduced susceptibility
		T821I	21	Ganciclovir reduced susceptibility
		A834P	6.4	Ganciclovir and cidofovir reduced susceptibility
		T838A	2.4	-
		G841A	4.3	Ganciclovir and cidofovir reduced susceptibility

Abbreviations: CMV, cytomegalovirus; EC_50_, half maximal effective concentration. Figure adapted from Lurain et al.^167^ and Cotton et al.^3^

**Figure 2:**
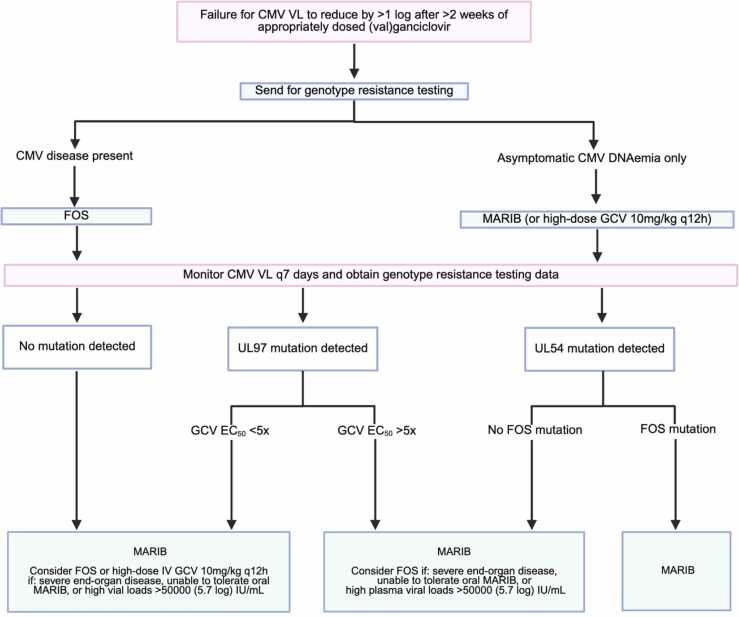
Management algorithm of post-transplant (val)ganciclovir resistant/refractory cytomegalovirus infection in thoracic organ transplant recipients. *Abbreviations: CMV, cytomegalovirus; EC*_*50*_*, drug concentration required to reduce viral growth by 50% in cell culture; FOS, foscarnet; GCV, ganciclovir; IU, international unit; IV, intravenous; MARIB, maribavir; VL, viral load.*

Suspected R/R infection should prompt genotypic resistance testing. For patients already receiving (val)ganciclovir, treatment options while awaiting resistance testing results include foscarnet or maribavir (Fig. 2). Foscarnet is active against most resistant strains but is limited by intravenous administration, nephrotoxicity, electrolyte derangement, and frequent need for hospitalization to coordinate initiation.

Maribavir, an oral UL97 kinase inhibitor, demonstrated superior sustained virologic clearance at 8 weeks compared to investigator-assigned therapy (IAT) in a phase 3 RCT involving 353 SOT and HSCT recipients (including 62 LTRs and 23 HTRs) with R/R infection.^97^ Most participants (65.1%) had low-level DNAemia <9100 IU/mL. In subgroup analyses, maribavir achieved its endpoint in LTRs (47.5% vs 13.6%, p<0.001) but not HTRs (42.9% vs 11.1%, p=0.063), although analyses were limited by small sample size (lung: n=40 maribavir vs n=22 IAT group, heart: n=14 maribavir vs n=9 IAT group).^98^ Subsequent observational data supports maribavir’s short-term effectiveness in TOTRs,^99^ however relapsed infection (often with maribavir resistance) during or after maribavir treatment remains common. As such, maribavir may be best suited for short-term (<8 weeks) therapy in R/R infections, where it is quickly stopped or changed to an alternative prophylaxis agent following viral suppression. Cost remains a major barrier to rapid maribavir access in outpatient settings.

### Applications of CMV-specific cell mediated immunity assays

**Figure 3:**
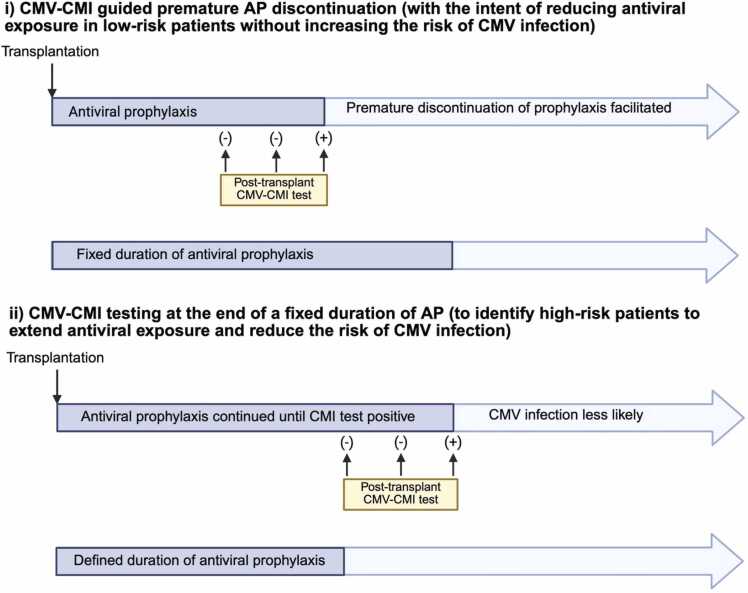

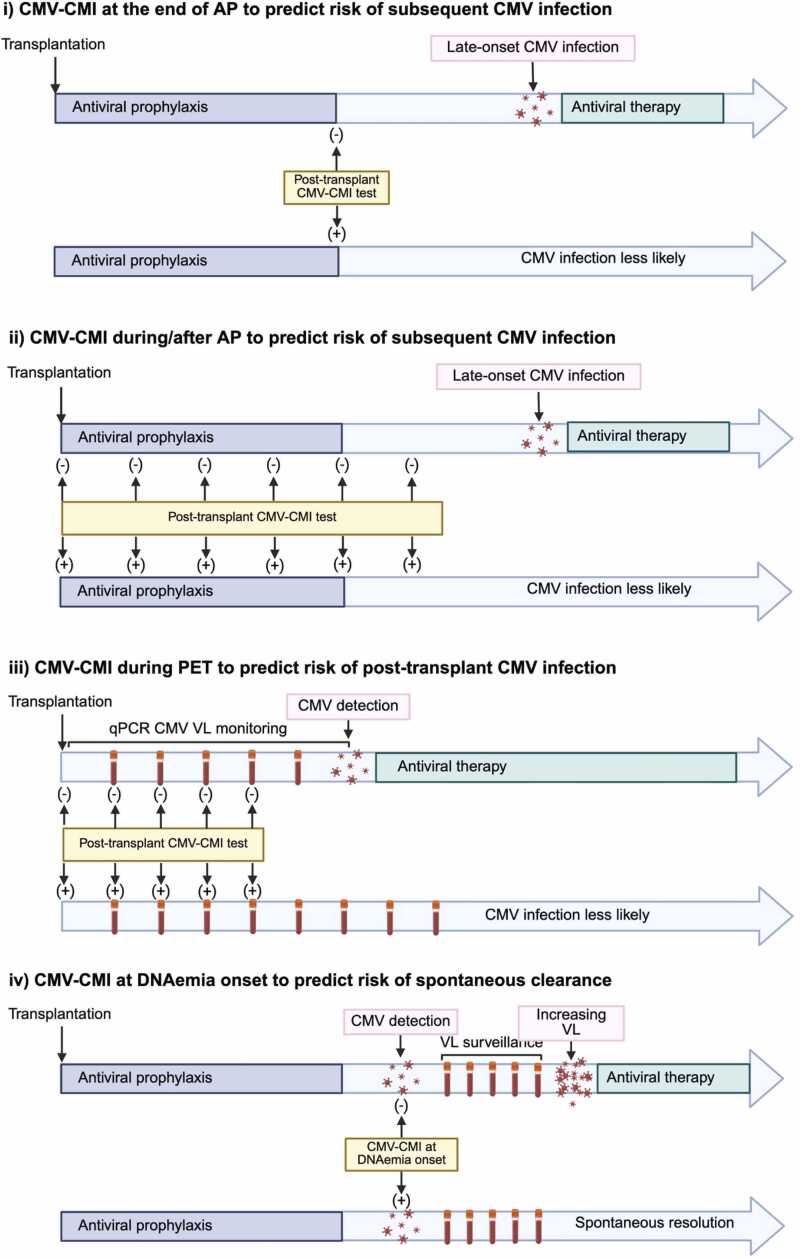

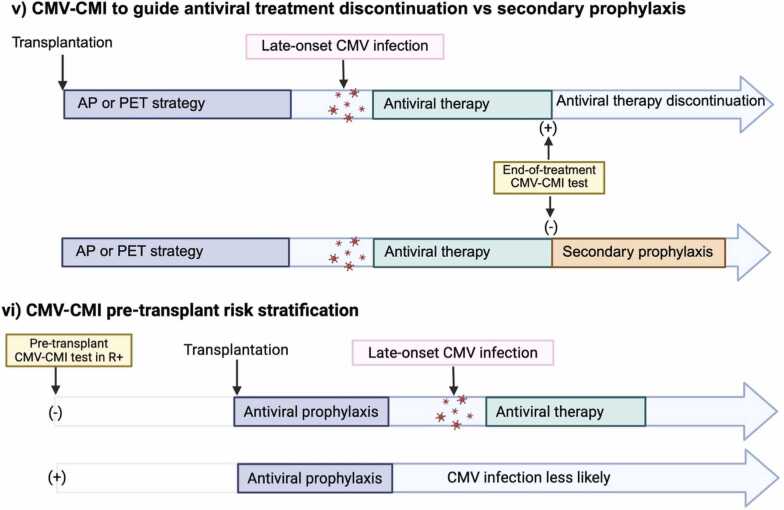
a. Clinical applications of cytomegalovirus cell-mediated immune (CMV-CMI) monitoring investigated in thoracic organ transplant recipients. *Abbreviations: AP, antiviral prophylaxis; CMI, cell-mediated immunity; CMV, cytomegalovirus*. b. Predictive value of cytomegalovirus cell-mediated immune (CMV-CMI) monitoring investigated in thoracic organ transplant recipients in research settings. *Abbreviations: AP, antiviral prophylaxis; CMI, cell-mediated immunity; CMV, cytomegalovirus; PET, pre-emptive therapy; qPCR, quantitative polymerase chain reaction; R, recipient; VL, viral loads*.

CMV-CMI is central to long-term CMV control following transplantation. Most commercially available CMV-CMI assays measure interferon-γ production to quantify cellular responses to CMV antigens.^100^ CMV-CMI has several potential applications (Fig. 3). Most studies describing performance characteristics of CMV-CMI assays in SOTRs were performed in kidney transplant recipients, however growing evidence describes potential applications in TOTRs (Table 5). Most studies in TOTRs have evaluated CMV-CMI as a tool for identifying individuals at risk of CMV DNAemia^101^, ^102^, ^103^, ^104^, ^105^, ^106^, ^107^, ^108^, ^109^, ^110^ or BAL detection,^103^, ^111^, ^112^ rather than CMV disease.^110^, ^113^, ^114^, ^115^

**Table 5:** Studies Assessing the Role of Cytomegalovirus Cell-mediated Immune Monitoring (CMV-CMI) in Thoracic Organ Transplant Recipients, Stratified by Timepoint of Assessment

Assessment Timepoint	Population	CMV Serostatus	Sample Size	CTR Sample Size	Study Design	Assay	Antigen Stimulation Positive Cutoff	Primary CMV Endpoint	Major Finding	Reference
CMI-guided premature AP discontinuation (Fig. 3a.i)	Lung	R+	150	150 LTRs, 0 HTRs.	Randomized noninferiority clinical trial evaluating the efficacy and safety of CMI guided AP discontinuation after 3 months of AP vs 6 months of AP.	QuantiFERON- CMV	IFN ≥ 0.2 IU/mL	Incidence of CMV disease up to 18 months post-transplant.	Incidence of CMV disease at 18 months did not differ between the CMI-guided AP discontinuation group and the 6 months AP group (18.7% vs 16.0%, p 0.62).	^113^
CMI-guided premature AP discontinuation (Fig. 3a.i)	Mixed SOT	D+R-, or R+ with ATG induction	89	0 LTRs, 2 HTRs.	Single-arm prospective interventional clinical trial using CMI at 3, 4, 5, and 6 months post-transplant to guide AP discontinuation before 6 months, in patients expected to receive a minimum of 3 months of AP.	QuantiFERON- CMV	IFN ≥ 0.2 IU/mL	Incidence of CMV DNAemia (>1000 IU/mL) up to 1 year post-transplant.	Significantly lower rates of CMV DNAemia >1000 IU/mL occurred in the CMI-positive vs CMI-negative patients (11.8% vs 38.2%, p 0.008).	^101^
CMI-guided AP prolongation (Fig. 3a.ii)	Heart	D+R-, R+, D-R-	154	0 LTRS, 154 HTRs.	Non-randomized clinical trial comparing CMI-guided AP discontinuation after day 100 vs clinician-directed discontinuation.	QuantiFERON- CMV	IFN ≥ 0.2 IU/mL	Incidence of CMV DNAemia (>137 IU/mL) up to 1 year post-intervention.	Significantly lower rates of CMV DNAemia occurred with CMI-guided AP discontinuation at day 100 than clinician-directed discontinuation (5% vs 19%, p 0.03).	^102^
CMI-guided AP prolongation (Fig. 3a.ii)	Lung	D+R-, R+	118	118 LTRs, 0 HTRs.	Randomized clinical trial comparing CMI-guided AP discontinuation 5-11 months post-transplant vs AP discontinuation at 5 months.	QuantiFERON- CMV	IFN ≥ 0.2 IU/mL	Incidence of CMV DNA detected in the lung allograft (BAL DNA > 600 copies/mL) up to 18 months post-transplant.	Significantly lower rates of CMV DNA detected in the lung allograft in the CMI-guided AP discontinuation group vs the 5 months AP group (37% vs 58%, p 0.03).	^111^
CMI-guided AP prolongation (Fig. 3a.ii)	Lung	D+R-, R+	263	263 LTRs, 0 HTRs.	Retrospective analysis of real-world experience with CMI-guided AP discontinuation at 5-11 months post-transplant vs AP discontinuation at 5 months.	QuantiFERON- CMV	IFN ≥ 0.2 IU/mL	Incidence of CMV infection (DNAemia or BAL CMV DNA ≥ 1000 IU/mL) up to 2 years post-transplant.	CMI-guided AP discontinuation was associated with a reduced risk of CMV infection when compared to the 5 months AP group (43% vs 60%, p <0.001).	^103^
CMI at end of AP (Fig. 3b.i)	Heart	R+	44	0 LTRs, 44 HTRs.	Retrospective analysis of a prospective observational cohort study evaluating CMI at 0.5, 1, 3, 6, and 12 months post-transplant in patients who received PET vs 3 months AP.	QuantiFERON- CMV	IFN ≥ 0.2 IU/mL	Incidence of CMV DNAemia (>103 IU/mL) detected in at least 2 consecutive samples up to 1 year post-transplant.	In the AP group, a significantly higher incidence of CMV DNAemia occurred in patients without detectable CMI at AP discontinuation (3 months post-transplant) compared to those with detectable CMI (66.7% vs 14.3% p 0.036).	^104^
CMI at end of AP (Fig. 3b.i)	Lung	R+	60	60 LTRs, 0 HRTS.	Retrospective analysis of CMI performed at end of AP in patients who received 6 months of AP.	T.SPOT.CMV	≥ 55 ELISPOTS	Incidence of CMV infection (detection of viral proteins or nucleic acid in any body fluid or tissue specimen) up to 1 year post AP discontinuation.	A significantly higher incidence of CMV infection occurred in patients classified as high risk (<55 ELISPOTS) by CMI when compared to patients considered low risk (≥55 ELISPOTS) (65.6% vs 35.7%, p 0.021).	^168^
CMI at end of AP (Fig. 3b.i)	Lung	D+R-	50	50 LTRs, 0 HTRs.	Retrospective analysis of CMI performed at a median of 5.5 months post-transplant in patients who received AP for 6-9 months.	T.Track CMV	>10 spots IE1 and/or >10 spots pp65	Incidence of CMV DNAemia (≥137 IU/mL) detected in 2 consecutive plasma PCR samples up to a median follow-up of 17.5 months.	Positive CMI prior to AP discontinuation was significantly associated with a lower odds of CMV DNAemia >3 months post-transplant (OR 0.05, p 0.01).	^105^
CMI at end of AP (Fig. 3b.i)	Lung	D+R-, R+	38	38 LTRs, 0 HTRs.	Prospective observational cohort of CMI performed at 0.5, 1.5, 3, 6, 9, and 12 months post-transplant in D+R- patients who received 6 months of daily AP and R+ patients who received 3 months of intermittent AP.	QuantiFERON- CMV	IFN ≥ 0.2 IU/mL	Incidence of CMV DNAemia (detection limit not specified) and CMV disease up to 2 years post-transplant.	CMI was able to predict CMV disease among R+ recipients at the end of AP (90 days; p 0.027) but was not able to predict CMV DNAemia at the same timepoint (90 days, p 0.265). CMI performance in D+R- was not reported.	^106^
CMI at end of AP (Fig. 3b.i)	Mixed SOT	D+R-, R+ with ATG induction	108	48 LTRs, 0 HTRs.	Prospective observational cohort of CMI performed at 0, 1, 2, and 3 months post-transplant in patients who received AP for 3 months.	QuantiFERON- CMV	IFN ≥ 0.1 IU/mL	Ability of CMI to predict CMV disease up to 6 months post-transplant.	CMI response at the end of 3 months of AP predicted the development of CMV disease (5.3% CMI positive group vs 22.9% CMI negative group, p 0.038).	^114^
CMI at end of AP (Fig. 3b.i)	Mixed SOT	D+R-	127	14 LTRs, 4 HTRs.	Prospective observational cohort of CMI performed at the end of AP and 1 and 2 months post AP discontinuation in patients who received AP for 3-6 months.	QuantiFERON- CMV	IFN ≥ 0.1 IU/mL	Incidence of CMV disease up to 1 year post-transplant.	Patients with a positive CMI result had a significantly lower incidence of CMV disease than patients with negative or intermediate CMI results (6.4% vs 22.2% vs 58.3%, p 0.001).	^115^
CMI during/after AP (Fig. 3b.ii)	Lung	D+R- R+, D-R-	67	67 LTRs, 0 HTRs.	Prospective observational cohort of CMI at various intervals post-transplant in D+R- patients who received AP for a mean of 318 days and R+ or D-/R- patients who received AP for a mean of 96 days.	QuantiFERON- CMV	IFN ≥ 0.2 IU/mL	Prediction of CMV DNAemia up to 1 year post-transplant.	CMI results differed significantly in patients who subsequently experienced episodes of high-level CMV DNAemia (>1000 copies/mL) and low plasma DNA levels (p 0.005).	^107^
CMI during/after AP (Fig. 3b.ii)	Lung	D+R-, R+, D-R-	39	39 LTRs, 0 HTRs.	Prospective observational cohort of CMI at 0.5, 1, 2, 3, 6, 9, 12, and 18 months post-transplant in patients who received AP for 5 months.	QuantiFERON- CMV	IFN ≥ 0.2 IU/mL	Incidence of CMV DNA detected in the lung allograft (BAL DNA > 400 copies/mL) up to a mean follow-up of 18 months post-transplant.	CMI at any time point did not significantly predict episodes of CMV DNA detection in the lung allograft.	^112^
CMI during PET (Fig. 3b.iii)	Heart	R+	48	0 LTRs, 48 HTRs.	Cross-sectional analysis of CMI performed within 100 days pre and/or post-transplant in patients who received PET.	ELISPOT	High responders (>100 spots), midresponders (51-99 spots), low responders (<50 spots)	Association between ELISPOT levels and the development of CMV DNAemia to 100 days post-transplant.	Patients protected from CMV DNAemia displayed significantly higher median ELISPOT levels than patients who developed CMV DNAemia (173 vs 18 spots, p NR)	^108^
CMI during PET (Fig. 3b.iii)	Heart	R+	44	0 LTRs, 44 HTRs.	Retrospective analysis of a prospective observational cohort study measuring CMI at 0.5, 1, 3, 6, and 12 months post-transplant in patients who received PET or AP for 3 months.	QuantiFERON- CMV	IFN ≥ 0.2 IU/mL	Incidence of CMV DNAemia (>103 IU/mL) detected in at least 2 consecutive samples up to 1 year post-transplant.	In the PET group, no significant difference in post-transplant CMV infection was observed in patients with and without detectable CMI at 0.5 months post-transplant (55.6% vs 66.7%, p 0.633).	^104^
CMI at asymptomatic DNAemia onset (Fig. 3b.iv	Mixed SOT	D+R-, R+	37	3 LTRs, 2 HTRs.	Prospective observational cohort of patients who had recent onset (<7 days) asymptomatic CMV DNAemia 50-15000 copies/mL.	QuantiFERON- CMV	IFN ≥ 0.2 IU/mL	Association between CMI at onset of asymptomatic CMV DNAemia and spontaneous viral clearance.	A positive CMI testat CMV infection onset was associated with higher rates of spontaneous clearance compared to a negative CMI (92.3% vs 45.5%, p 0.004).	^169^
CMI at end of antiviral treatment (Fig. 3b.v)	Mixed SOT	D+R-, R+, D-R-	27	6 LTRs, 0 HTRs.	Single-arm prospective interventional trial using CMI at the end of treatment to guide antiviral discontinuation vs 2 months of secondary antiviral prophylaxis.	QuantiFERON- CMV	IFN ≥ 0.2 IU/mL	Incidence of CMV DNAemia recurrence (≥500 IU/mL) and re-initiation of antiviral treatment up to 6 months.	Significantly lower rates of CMV DNAemia recurrence occurred in CMI-positive patients at the end of antiviral treatment discontinuation compared to CMI-negative patients (7.1% vs 69.2%, p 0.001).	^109^
CMI pre-transplant (Fig. 3b.vi)	Lung	R+ (59%) R- (41%)	39	39 LTRs, 0 HTRs.	Prospective observational cohort of CMI performed pre-transplant.	QuantiFERON- CMV	IFN ≥ 0.2 IU/mL	Incidence of CMV reactivation (not otherwise specified) and CMV disease up to 5 years post-transplant.	The incidence of CMV reactivation was significantly higher in CMI negative compared to CMV positive patients (HR 4.28; 95% CI, 1.16-15.76, p 0.01).	^110^

White rows indicate studies containing only cardiothoracic recipients. Gray rows indicate studies containing a subgroup of cardiothoracic recipients within a larger cohort of solid organ transplant recipients.

Abbreviations: AP, antiviral prophylaxis; ATG, anti-thymocyte globulin; BAL, bronchoalveolar lavage; CI, confidence interval; CMI, cell-mediated immunity; CMV, cytomegalovirus; D, donor; DNA, deoxyribonucleic acid; HTR, heart transplant recipient; HR, hazard ratio; IFN, interferon; IU, international units; LTR, lung transplant recipient; mL, milliliters; NR, not reported; PET, pre-emptive therapy; R, recipient; SOT, solid organ transplant.

#### CMV-CMI to guide duration of prophylaxis

The most well-established use of CMV-CMI testing in TOTRs is to individualize duration of AP rather than relying on a fixed duration (Table 5**,** Fig. 3a.i and 3a.ii). Other applications include CMV-CMI to guide management at onset of asymptomatic DNAemia to predict spontaneous resolution (Table 5**,** Fig. 3b.iv) and at end of treatment of CMV infection (Table 5**,** Fig. 3b.v).

Studies comparing CMV-CMI monitoring to fixed AP durations have considered two different applications of CMV-CMI. First, CMV-CMI monitoring may be used during prophylaxis to guide early discontinuation in individuals with positive CMV-CMI, with the intent of reducing antiviral exposure without increasing the risk of CMV infection (Table 5**,** Fig. 3a.i). This strategy is best studied in CMV seropositive kidney recipients.^101^ Second, CMV-CMI testing at the end of a fixed duration of prophylaxis can identify individuals who remain at high-risk for CMV infection, thereby offering extended prophylaxis to select patients with the intent of reducing CMV infections (Table 5**,** Fig. 3a.ii). The latter has been the focus of CMV-CMI monitoring studies in TOTRs.

CMV-CMI testing tends to have lower predictive value for CMV infections in D+R- SOTRs receiving prophylaxis.^116^ The only RCT to include D+R- LTRs evaluated CMI-guided AP discontinuation between 5- and 11-months post-transplant versus a fixed 5-month regimen and found that CMI-guided discontinuation was associated with reduced incidence of CMV DNA >600 copies/mL in BAL fluid.^111^ However, this primary endpoint has unclear clinical relevance, and the trial’s relatively short prophylaxis period (5 months instead of the current 12-month recommendation in D+R- LTRs) further limits interpretability.

#### Limitations of CMV-CMI assays

While CMV-CMI testing shows promise in guiding AP discontinuation in select seropositive TOTRs, data in liver and kidney transplant recipients suggest the predictive value remains poor in D+R- patients who experience the greatest morbidity from CMV infections. Further, it remains unclear whether CMV-CMI testing adds predictive capabilities beyond readily available and inexpensive clinical data, such as serostatus, immunosuppression intensity, and lymphopenia. “Logistics” is often cited as a barrier to PET implementation, and CMV-CMI monitoring to individualize AP duration would have similar logistical challenges. Future studies should assess CMV-CMI as a practical mechanism for directing antivirals toward high-risk patients and away from low-risk patients.

## Future of CMV prevention and management in TOTRs

As cardiothoracic transplant technologies evolve, CMV prevention must also advance in parallel. Innovative techniques, such as ex-vivo lung perfusion, are in their infancy and represent potential opportunities to study or mitigate allograft-derived CMV. Xenotransplantation and associated novel immunomodulators will provide important insights on immune control of human and non-human CMV.

TOTRs face high CMV-related morbidity yet remain underrepresented in clinical research. Investigational approaches, including CMV vaccines, virus-specific T-cells, and monoclonal antibodies, are under evaluation with most clinical trials targeting kidney and liver transplant recipients.^116^, ^117^ Inclusion of TOTRs in studies assessing novel interventions is necessary to understand their utility specifically in TOTRs and ensure equitable access to potential benefits of these investigative approaches. While recent progress is encouraging, dedicated research and novel prevention strategies will be essential to improving outcomes in this vulnerable population.

## Funding

This research did not receive any specific grant from funding agencies in the public, commercial, or not-for-profit sectors.

## CRediT authorship contribution statement

B.J.C.: writing – original draft, writing – review and editing. M.R.H.: supervision, writing – review and editing. C.R.W.: supervision, writing – review and editing.

## Declaration of Competing Interest

The authors declare that they have no known conflicts that could have appeared to influence the work reported in this paper.
